# *Lactobacillus hayakitensis* sp. nov., isolated from intestines of healthy thoroughbreds

**DOI:** 10.1099/ijs.0.65135-0

**Published:** 2007-12

**Authors:** Hidetoshi Morita, Chiharu Shiratori, Masaru Murakami, Hideto Takami, Yukio Kato, Akihito Endo, Fumihiko Nakajima, Misako Takagi, Hiroaki Akita, Sanae Okada, Toshio Masaoka

**Affiliations:** 1 School of Veterinary Medicine, Azabu University, 1-17-71 Fuchinobe, Sagamihara, Kanagawa 229-8501, Japan; 2 Microbial Genome Research Group, Japan Agency of Marine-Earth Science and Technology, 2-15 Natsushima, Yokosuka, Kanagawa 237-0061, Japan; 3 Nodai Culture Collection Center, Department of Brewing, Tokyo University of Agriculture, 1-1-1 Sakuragaoka, Setagaya, Tokyo 156-8502, Japan; 4 Northern Farm, 275 Hayakita-genbu, Abira-cho, Yufutsu-gun, Hokkaido 059-1432, Japan; 5 Clossfield-Bio Inc., 1-1-20, Higashi Nihonbashi, Chuo, Tokyo 103-0004, Japan

## Abstract

Two strains, KBL13^T^ and GBL13, were isolated as one of intestinal lactobacilli from the faecal specimens from different thoroughbreds of the same farm where they were born in Hokkaido, Japan. They were Gram-positive, facultatively anaerobic, catalase-negative, non-spore-forming and non-motile rods. KBL13^T^ and GBL13 homofermentatively metabolize glucose, and produce lactate as the sole final product from glucose. The 16S rRNA gene sequence, DNA–DNA hybridization, DNA G+C content and biochemical characterization indicated that these two strains, KBL13^T^ and GBL13, belong to the same species. In the representative strain, KBL13^T^, the DNA G+C content was 34.3 mol%. *Lactobacillus salivarius* JCM 1231^T^ (=ATCC 11741^T^; AF089108) is the type strain most closely related to the strain KBL13^T^ as shown in the phylogenetic tree, and the 16S rRNA gene sequence identity showed 96.0 % (1425/1484 bp). Comparative 16S rRNA gene sequence analysis of this strain indicated that the two isolated strains belong to the genus *Lactobacillus* and that they formed a branch distinct from their closest relatives, *L. salivarius*, *Lactobacillus aviarius*, *Lactobacillus saerimneri* and *Lactobacillus acidipiscis*. DNA–DNA reassociation experiments with *L. salivarius* and *L. aviarius* confirmed that KBL13^T^ represents a novel species, for which the name *Lactobacillus hayakitensis* sp. nov. is proposed. The type strain is KBL13^T^ (=JCM 14209^T^=DSM 18933^T^).

Lactobacilli are important members of healthy gastrointestinal tracts of mammals and humans, and some of them are frequently administered as probiotics for their beneficial roles in mammalian and human health. However, there have only been a few studies on the *Lactobacillus* flora of thoroughbred gastrointestinal contents by using the culturing method (Mitsuoka & Kaneuchi, 1977; Morotomi *et al.*, 2002). In our study, we isolated the following lactobacilli present in the intestinal tract of healthy thoroughbreds: *Lactobacillus gasseri*, *Lactobacillus johnsonii*, *Lactobacillus ruminis*, *Lactobacillus reuteri*, *Lactobacillus salivarius*, *Lactobacillus crispatus* and *Lactobacillus agilis*. These species are well-known species isolated from mammalian gastrointestinal tracts. *Lactobacillus equi* was also found during our study, which is a dominant and indigenous species in equine gastrointestinal tracts (Morotomi *et al.*, 2002). As part of a study on the intestinal microbiota in thoroughbreds, two strains, KBL13^T^ and GBL13, were isolated from different thoroughbreds. A polyphasic taxonomic study of these strains was performed using phenotypic characterization and phylogenetic as well as genetic methods; the results obtained by using these methods consistently revealed the isolates, KBL13^T^ and GBL13, to represent a novel *Lactobacillus* species, from intestines of thoroughbreds, for which the name *Lactobacillus hayakitensis* sp. nov. is proposed.

Bacterial strains, KBL13^T^ and GBL13, were isolated from fresh faeces of different healthy thoroughbreds of the same farm where they were born in Hokkaido, Japan. The fresh faeces of each thoroughbred were transferred under anaerobic conditions by AnaeroPack (Mitsubishi Gas Chemical) at 4 °C to our laboratory within 24 h. The initial processing and subsequent weighing and dilution of the specimens were carried out under anaerobic conditions. Each dilution was then spread on to BL agar plates (Eiken Chemical) and incubated anaerobically at 37 °C for 2 days. All further cultivation was performed at 37 °C in ABCM broth (Eiken Chemical). The 16S rRNA gene sequences of the isolates were determined as described previously (Endo & Okada, 2005). The 1484 bp of the 16S rRNA gene sequence of KBL13^T^ was consistent with those of GBL13. DNA–DNA hybridization was carried out by using the microdilution-well technique, with photobiotin for labelling of the DNA (Ezaki *et al.*, 1989). KBL13^T^ and GBL13 shared high levels of DNA–DNA relatedness (99.5–100.0 %). The closest known relatives of the isolates were determined by performing database searches, and the sequences of closely related species were retrieved from the DDBJ database. Multiple alignments of the sequences were carried out with the clustal_x program, version 1.18 (Thompson *et al.*, 1997). Distance matrices for the aligned sequences were calculated by using the two-parameter method of Kimura (1980). The neighbour-joining method was used to construct a phylogenetic tree (Saitou & Nei, 1987). The robustness of individual branches was estimated by using bootstrapping with 1000 replicates (Felsenstein, 1985). Phylogenetic trees were also constructed by using the maximum-likelihood (Cavalli-Sforza & Edwards, 1967) and maximum-parsimony (Kluge & Farris, 1969) methods with phylip version 3.65 (Felsenstein, 2005).

In a neighbour-joining dendrogram created based on the sequence of KBL13^T^ and sequences from the GenBank database, the phylogenetic position of KBL13^T^ was determined. KBL13^T^ was placed within the *L. salivarius* phylogenetic group (Canchaya *et al.*, 2006) and was most closely related to *L. salivarius*, *Lactobacillus* *aviarius*, *Lactobacillus* *saerimneri* and *Lactobacillus* *acidipiscis* as shown in Fig. 1. Recently, on the basis of a polyphasic analysis, Li *et al.* (2006) indicated that *L. salivarius* subsp. *salivarius* and *L. salivarius* subsp. *salicinicus* did not merit separate subspecies status. As the information of the physiological characteristics of *L. salivarius* JCM 1150 is available to us (previously described as *L. salivarius* subsp. *salicinicus* JCM 1150), the physiological characteristics of KBL13^T^ and GBL13 were compared with those of *L. salivarius* JCM 1231^T^ (=ATCC 11741^T^; AF089108) and JCM 1150 as shown in Table 1. *L. salivarius* JCM 1231^T^ and JCM 1150, *L. aviarius* subsp. *aviarius* JCM 5666^T^ and *L. aviarius* subsp. *araffinosus* JCM 5667^T^ used in the study were obtained from the Japan Collection of Microorganisms. A high similarity of 96.0 % (1425/1484 bp) was observed in the 16S rRNA gene sequences of KBL13^T^ and *L. salivarius* JCM 1231^T^. Identical tree topologies were obtained by using the maximum-likelihood and maximum-parsimony methods (see Supplementary Figs S1 and S2 available in IJSEM Online).

The DNA G+C content was determined by hydrolysing the DNA enzymically and quantifying the nucleosides by HPLC according to the method of Ezaki *et al.* (1990). The DNA G+C content of KBL13^T^ and GBL13 were 34.3 and 34.8 mol%, respectively. The DNA G+C content of their closest relatives, *L. salivarius* JCM 1231^T^ and *L. aviarius* subsp. *araffinosus* JCM 5667^T^ was 34.7 and 41.3 mol%, respectively. The DNA G+C content of KBL13^T^ was found to be within the range of 32.0–55.0 mol%, which is the range reported for *Lactobacillus* species (Kandler & Weiss, 1986).

The sugar fermentation patterns were determined using the API 50CH system (bioMérieux) according to the manufacturer's instructions. The results were recorded after 48 h at 37 °C. The isomer of lactic acid produced from glucose was determined by using an F-kit (d-lactic acid/l-lactic acid; Roche Diagnostics Corporation). Other biochemical tests, such as those on motility, growth at a fixed temperature and gas production from glucose, were performed by using the methods described by Mitsuoka (1969). Table 1 shows the characteristics most useful in distinguishing the strains studied from closely related lactobacilli. Since KBL13^T^ and GBL13 were found to be the same species, KBL13^T^ was used as a representative strain in the experiments described below.

DNA–DNA hybridization analyses (Ezaki *et al.*, 1989) were performed, including those for the two most closely related species, *L. salivarius* JCM 1231^T^ and JCM 1150, and *L. aviarius* subsp. *araffinosus* JCM 5667^T^, based on the 16S rRNA gene sequence analysis. DNA–DNA relatedness values between KBL13^T^ and *L. salivarius* JCM 1231^T^ and JCM 1150, and *L. aviarius* subsp. *araffinosus* JCM 5667^T^ were 14.2, 12.1 and 7.9 %, respectively. These values are well below the threshold of 70.0 % that is suggested for species delineation (Stackebrandt & Goebel, 1994), indicating that strain KBL13^T^ represents a separate genomic species. Analysis by high-performance thin-layer chromatography showed that *meso*-diaminopimelic acid was not contained in the peptidoglycan of the strain KBL13^T^, and an analysis, by ultraperformance liquid chromatography according to the methods described by Komagata & Suzuki (1987), of the cell wall composition revealed the Lys–Asp peptydoglycan type in the presence of Lys, Glu, Ala and Asp.

DNA–DNA relatedness showed a clear separation of strain KBL13^T^ from its phylogenetic relatives, it is considered that the strain studied represents a novel species belonging to the genus *Lactobacillus*, for which the name *Lactobacillus hayakitensis* sp. nov. is proposed.

## Description of *Lactobacillus hayakitensis* sp. nov.

*Lactobacillus hayakitensis* (ha.ya.ki.ten′sis. N.L. masc. adj. *hayakitensis* of Hayakita, which is the name of the area where the bacterium was originally isolated).

Cells are Gram-positive, 3.0–5.0 μm long and 1.0–1.5 μm wide, non-motile and non-spore-forming rods. They occur singly or in pairs. Colonies are small (1.5 mm), circular to slightly irregular, convex, with a smooth to rough surface, and white when grown on MRS agar. The optimum growth temperature is 37 °C. Strain KBL13^T^ is not able to grow in 4.5 % NaCl and at 15 °C, but grows in 3.0 % NaCl and at 45 °C. Cells are catalase-negative. Glucose is metabolized homofermentatively and lactate is the sole final product. Strain KBL13^T^ produces l(+)-lactic acid. Acid is produced from glucose, fructose, mannose, mannitol, *N*-acetyl-d-glucosamine, arbutin, aesculin, salicin, cellobiose, maltose, sucrose and gentiobiose. Amygdalin and raffinose are weakly fermented. In this species, some strains cannot ferment *N*-acetyl-d-glucosamine, arbutin and raffinose. The DNA G+C content of the type strain is 34.3 %, and the cell wall composition of the strain exhibits the Lys–Asp peptydoglycan type. 

The type strain, KBL13^T^ (=JCM 14209^T^=DSM 18933^T^), was isolated from the faeces of a thoroughbred.

## Figures and Tables

**Fig. 1. f1:**
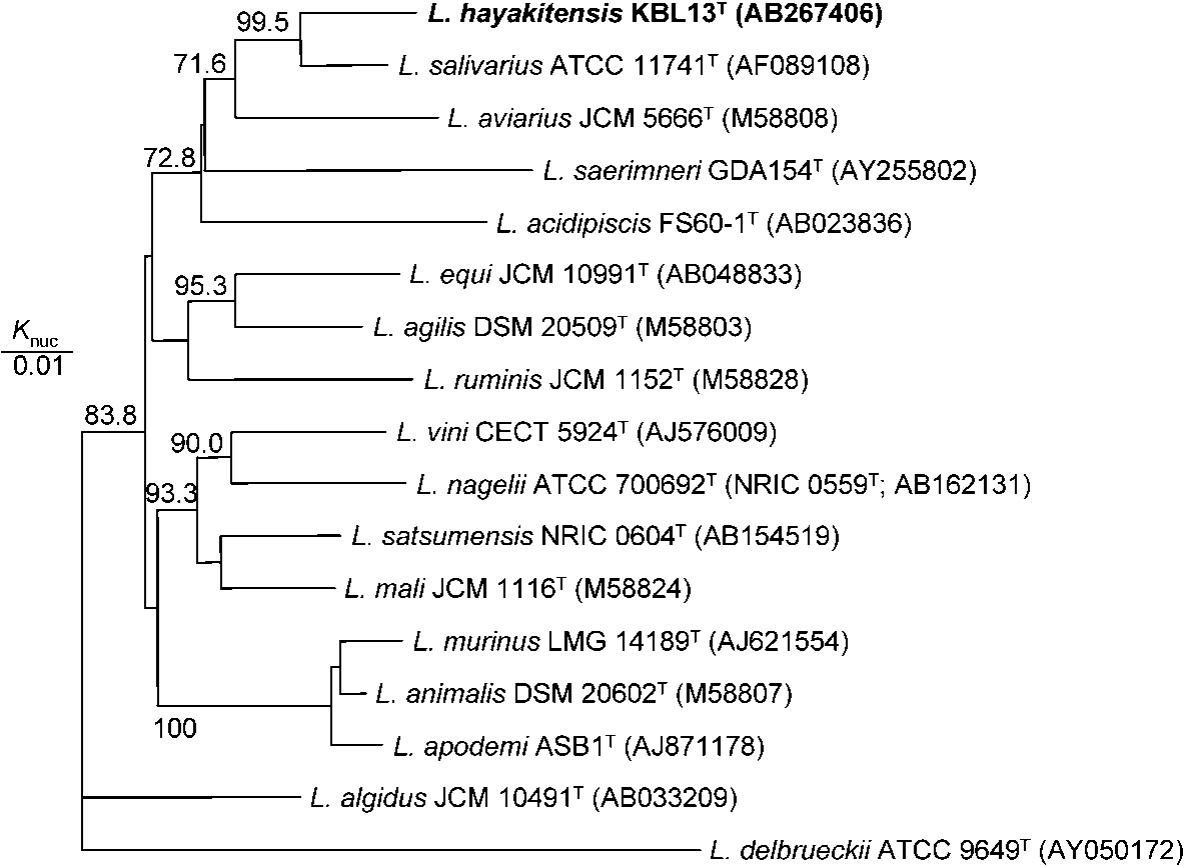
Phylogenetic relationship of the isolate to the species of the *L. salivarius* phylogenetic group based on the 16S rRNA gene sequences is shown. The tree was constructed by the neighbour-joining method. *L. delbrueckii* ATCC 9649^T^ was used as an outgroup. Bootstrap percentages above 70.0 % are given at the branching points.

**Table 1. t1:** Physiological characteristics of strains KBL13^T^ and GBL13 and type strains of the closely related *Lactobacillus* species Strains: 1, KBL13^T^; 2, GBL13; 3, *L. salivarius* JCM 1231^T^; 4, *L. salivarius* JCM 1150; 5, *L. aviarius* subsp. *aviarius* JCM 5666^T^ (Fujisawa *et al.* 1984); 6, *L. aviarius* subsp. *araffinosus* JCM 5667^T^ (Fujisawa *et al.* 1984). +, Positive; –, negative; w, weakly positive; nd, no data available. All strains were positive for the following characteristics: fermentation of glucose, fructose, mannose, maltose, sucrose; growth in MRS broth at 37 °C and no growth in MRS broth at 15 °C. The DNA G+C contents were determined by HPLC.

**Characteristic**	**1**	**2**	**3**	**4**	**5**	**6**
Lactic acid isomers	l	l	Mainly l	Mainly l	dl	dl
Fermentation of:						
Galactose	–	–	+	+	+	–
Rhamnose	–	–	+	–	nd	nd
Sorbitol	–	–	+	+	nd	nd
*N-*Acetyl-d-glucosamine	+	–	+	+	nd	nd
Amygdalin	w	w	–	–	–	–
Arbutin	+	–	–	+	nd	nd
Aesculin	+	+	–	+	+	–
Salicin	+	+	–	+	+	–
Cellobiose	+	+	–	–	+	–
Lactose	–	–	+	+	–	–
Melibiose	–	–	+	+	+	–
Trehalose	–	–	+	+	+	+
Raffinose	w	–	+	+	+	–
Gentiobiose	+	+	–	–	nd	nd
Growth on MRS medium at 45 °C	+	+	+	+	nd	nd
DNA G+C content (mol%)	34.3	34.8	34.7	nd	38.7	41.3
Peptidoglycan type	Lys–Asp	Lys–Asp	Lys–Asp	Lys–Asp	Lys–Asp	Lys–Asp
